# Supplementary material for: Curated and harmonised transcriptomics datasets of interstitial lung diseases
Source: Data Brief. 2025 Oct 14;63:112139. doi: 10.1016/j.dib.2025.112139 (PMC12581653; doi:10.1016/j.dib.2025.112139)
Supplement: Supplementary file 1 [file mmc1.zip › Supplementary_material/RNA-seq/fastQC reports/GSE124685/SRR8397230_fastqc.html]

SRR8397230.fastq.gz FastQC Report 

FastQC Report

Tue 30 Jan 2024  
SRR8397230.fastq.gz

## Summary

- Basic Statistics
- Per base sequence quality
- Per sequence quality scores
- Per base sequence content
- Per sequence GC content
- Per base N content
- Sequence Length Distribution
- Sequence Duplication Levels
- Overrepresented sequences
- Adapter Content

## Basic Statistics

| Measure | Value |
| --- | --- |
| Filename | SRR8397230.fastq.gz |
| File type | Conventional base calls |
| Encoding | Sanger / Illumina 1.9 |
| Total Sequences | 9402855 |
| Sequences flagged as poor quality | 0 |
| Sequence length | 8-368 |
| %GC | 53 |

## Per base sequence quality

## Per sequence quality scores

## Per base sequence content

## Per sequence GC content

## Per base N content

## Sequence Length Distribution

## Sequence Duplication Levels

## Overrepresented sequences

| Sequence | Count | Percentage | Possible Source |
| --- | --- | --- | --- |
| GGGCCACGAGCTGAGTGCGTCCTGTCACTCCACTCCCATGTCCCTTGGGA | 333883 | 3.5508683266943923 | No Hit |
| AAGCTCAGGGAGAGCCCTGTTAGGGCCGCCTCTGGCCCTAGTCTCAGACC | 174392 | 1.854670735643589 | No Hit |
| AAACTCAGGGAGAGCCCTGTTAGGGCCGCCTCTGGCCCTAGTCTCAGACC | 105005 | 1.1167352894413451 | No Hit |
| CTTCCGCATAGCTGCTGTGGTCAAAAAGGAGCCCAGAGTGACAGTTTTCC | 74832 | 0.7958433901192776 | No Hit |
| GCTGCTACAGGAGAATAGCAGACAGGTATAGTTAAGAAACAACTTTATTT | 53982 | 0.5741022274617656 | No Hit |
| GGGCCACGAGCTGAGTGCGTCCTGTCACTCCACTCCCCATGTCCCTTGGG | 49949 | 0.5312109992124732 | No Hit |
| ACAAGCAGTTCACAGTAGAACACTGCAGACTGCAGGGCATTTTCAGGCTA | 47344 | 0.5035066477149759 | No Hit |
| CTAAGTGCTGTGTTGTCGTTCCCCCTGCTTAAAATAAAGTTGTTTCTTAA | 47081 | 0.5007096248958428 | No Hit |
| AGAACGGCGACCGTCAAGGAAAACTGTCACTCTGGGCTCCTTTTTGACCACAGCAGCTATGCGGAAGCAGCTGC | 45027 | 0.47886519573044567 | No Hit |
| AAGACCAACGTCAAGGCCGCCTGGGGTAAGGTCGGCGCGCACGCTGGCGA | 43034 | 0.4576695056980034 | No Hit |
| TCTCTGGGCTTCTATTTCGACCGCGATGATGTGGCTCTGGAAGGCGTGAG | 34900 | 0.37116386459219036 | No Hit |
| GGGAATGTCTACGTGCGTATGCACGTGGCACTCTCTGCCCGAGGTCCGGG | 34137 | 0.3630493078963783 | No Hit |
| TTTACACCTGCTAGGGTGTTCAAAGGTCAGTGCTATAGAAATTCAGTATC | 33258 | 0.35370108334117667 | No Hit |
| ACACTGAGGACTCTGTTCCTCCCCTTTCCGCCTAGGGGAAAGTCCCCGGA | 32874 | 0.34961721732388723 | No Hit |
| GGGGCCACGAGCTGAGTGCGTCCTGTCACTCCACTCCCATGTCCCTTGGG | 27789 | 0.2955378977980624 | No Hit |
| AAAGCCAAGAAAACCAACGATGCCAGATACTGAATTTCTATAGCACTGAC | 26283 | 0.27952148576150543 | No Hit |
| GAGCAGGGCAAGGCCTCAAACCGTTTCAAAACTGTTCTAGCAAGCACTGA | 22131 | 0.23536468444956346 | No Hit |
| TCAGTTACAACAAACAGAACGGCGACCGTCAAGGAAAACTGTCACTCTGG | 21982 | 0.23378005935431315 | No Hit |
| TGGTTCCTGGCAAGCCCATGTGTGTTGAGAGCTTCTCAGACTATCCACCT | 20644 | 0.21955033870032026 | No Hit |
| TTGGTGGTGGGGAAGGACAGGAACATCCTCTCCAGGGCCTCCGCACCATA | 20494 | 0.2179550785373166 | No Hit |
| CGGTGACCCTATGCATTCCTTCAGTGCTTGCTAGAACAGTTTTGAAACGG | 19807 | 0.2106487869907597 | No Hit |
| ACAAGCAGTTCACAGTAGAACACTGCAGACTGCAGGGCATTTTCTGGCTA | 17809 | 0.18939992161955066 | No Hit |
| GGGTACGGACCTCAGCTGAGTCATGGGAGCTGAATGTATGTGTTTCTCCT | 17514 | 0.18626257663231008 | No Hit |
| ACACTGAGGACTCTGTTCCTCCCCCTTTCCGCCTAGGGGAAAGTCCCCGG | 16140 | 0.17164999353919633 | No Hit |
| ACGCTCGTAGCCCTCGCGCTTCTCCTCGGCCAATTCGCGGAAGAAGTGGC | 15380 | 0.16356734204664433 | No Hit |
| GTGGTGAGGCCCTGGGCAGGCTGCTGGTGGTCTACCCTTGGACCCAGAGG | 15114 | 0.16073841402425115 | No Hit |
| CCACCTTCTCCATCGGCTCCACTGGCCTCGTGGTGTATGACTACCAGCAG | 15110 | 0.1606958737532377 | No Hit |
| CGCTGCACTGACCTTCTTCCAAGCCTCAGTTCCTGTTCTAGGAACTTGAG | 14990 | 0.15941966562283477 | No Hit |
| ACGCTCGTAGCCCTCGCGCTTCTCCTCGGCCAGTTCGCGGAAGAAGTGGC | 14813 | 0.15753725863049042 | No Hit |
| GGGGCCACGAGCTGAGTGCGTCCTGTCACTCCACTCCCCATGTCCCTTGG | 14604 | 0.15531452947003863 | No Hit |
| TTTCTTATAGCGGTGGTTTAAACAGAGGTGCAAACAGCAAGCGGATCTTG | 14379 | 0.1529216392255331 | No Hit |
| AAAAGTGATGGACTGCCCATTGCTGGAGAAGACCTTCTCTCCTACTGTCA | 13706 | 0.14576423862752325 | No Hit |
| AGCGGTTCCGCTGCCCTGAGGCACTCTTCCAGCCTTCCTTCCTGGGCATG | 13371 | 0.14220149093014833 | No Hit |
| ACACCCACCGCAACTGTCTGTCTCATATCACGAACAGCAAAGCGACCCAA | 13297 | 0.14141449591639987 | No Hit |
| TCACACTTCATGATGGAGTTGAAGGTAGTTTCGTGGATGCCACAGGACTC | 13209 | 0.14047860995410438 | No Hit |
| GGTTATAATCATTGGCAATGTTGTAATCAACAACCCATGTTATTTCTACA | 12392 | 0.13178975959961095 | No Hit |
| CCCATAACAGCATCAGGAGTGGACAGATCCCCAAAGGACTCAAAGAACCT | 12198 | 0.12972655645545955 | No Hit |
| AAGCTCAGGGAGAGCCCTGTTAGGGGCCGCCTCTGGCCCTAGTCTCAGAC | 11631 | 0.1236964730393056 | No Hit |
| AGTGTGGCGATTCCTCAGGGATCTAGAACTAGAAATACCATTTGACCCAG | 11619 | 0.12356885222626532 | No Hit |
| TGACTGGTGATGCTGATATTGGAGTGCATCTGAGGCTGTTGTAGAAATAA | 11371 | 0.12093135542343256 | No Hit |
| ATTCTTCCTACCCATGAGCATGGAATGTTCTTCCATTTGTTTGTATCCTC | 11249 | 0.1196338771575229 | No Hit |
| AAACTCAGGGGAGAGCCCTGTTAGGGCCGCCTCTGGCCCTAGTCTCAGAC | 10972 | 0.11668796338984277 | No Hit |
| AAAACAGCGTCTTACTGGGGAGGGATATGTGCTTTCTCATCTATCCTCAG | 10398 | 0.11058343449941535 | No Hit |
| TCAGAAAGGTCACGTCCTGCTCTTGGTCCTTGTCAGTGCCATGTTCTGTG | 10363 | 0.11021120712804781 | No Hit |
| CAGCTGGAGAAGGACTTCAGCAGCATGAAGAAGTACTGCCAAGTCATCCG | 9792 | 0.10413858344088045 | No Hit |
| CTAAGTGCTGTGTTGTCGTTCCCCCCTGCTTAAAATAAAGTTGTTTCTTA | 9580 | 0.10188394907716858 | No Hit |

## Adapter Content

Produced by FastQC (version 0.11.7)
